# Comparison of physical and psychological health outcomes for motorcyclists and other road users after land transport crashes: an inception cohort study

**DOI:** 10.1186/s12889-021-12003-0

**Published:** 2021-11-02

**Authors:** Lisa N. Sharwood, Annette Kifley, Ashley Craig, Bamini Gopinath, Jagnoor Jagnoor, Ian D. Cameron

**Affiliations:** 1 John Walsh Centre for Rehabilitation Research, University of Sydney, Northern Clinical School, Faculty of Medicine and Health, 1 Reserve Road, St Leonards, NSW 2065 Australia; 2 University of Technology Sydney, Faculty of Engineering and Risk, Ultimo, Australia; 3 John Walsh Centre for Rehabilitation Research, Kolling Institute, University of Sydney, Northern Clinical School, Faculty of Medicine and Health, 1 Reserve Road., St Leonards, NSW 2065 Australia; 4 The George Institute for Global Health, University of New South Wales, Sydney, Australia

**Keywords:** Motorcycle, Road traffic crash, Injury, Psychological outcomes, Injury prevention, Cohort, Bicyclists, Vehicle occupants, Quality of life

## Abstract

**Background:**

Serious injuries and fatalities among vulnerable road users on two wheeled motorised vehicles have increased across Australia and internationally in the past decade yet fallen for motor vehicle occupants. Almost half of all reported motorcycle injury crashes cause serious injury or death, nearly double that of motor vehicle police-reported crashes. This study explores associations with sociodemographic and pre-injury health characteristics and health outcomes after a road traffic injury; aiming to compare motorcyclists with other road users and inform recovery care.

**Methods:**

An inception cohort study recruited 1854 individuals aged > 17 years, injured following land-transport crashes in New South Wales, Australia (July 2013–November 2016). Interviews conducted at baseline, 6-and 12-months post-injury elicited demographic, socioeconomic, and self-reported health conditions.

**Results:**

Primary analysis involved 1854 participants who were recruited at baseline as three distinct road user groups; 628 (33.9%) motorcyclists, 927 (50%) vehicle occupants and 299 (16.1%) bicyclists. At baseline, injury patterns differed significantly between road user groups; motorcyclists were more than twice as likely to sustain lower extremity injury (*p* < 0.001); to have more severe injury severity scores (*p* < 0.001) and longer hospital stays versus vs vehicle occupants and bicyclists (< 0.001) across these measures. Injured motorcyclists were predominantly male (88.1%, *p* < 0.001), were younger on average (38 years) than bicyclists (41.5 years), had lower income and education levels, and poorer pre-injury physical health than other road user groups. Despite these differences, at 12 months post-injury motorcyclists had better physical health (SF12-PCS 2.07 (0.77, 3.36), *p* = 0.002) and reported lower pain scores (− 0.51 (− 0.83, − 0.2), *p* < 0.001) than vehicle occupants. Motorcyclists displayed less evidence of psychological distress than vehicle occupants, but more than bicyclists across several measures used.

**Conclusions:**

Road user types differ in important characteristics, including pre-injury health status and recovery after injury. As vulnerable road users experiencing transport crash and considering their higher initial injury severity, the degree of recovery among motorcyclists compared with other user types is remarkable and unexplained. Health and recovery outcomes after land-transport crashes is least favourable among vehicle occupants despite their higher levels of protection in a crash. This information is valuable for targeting early intervention strategies by road user type during the post-crash care phase, to improve long-term recovery.

**Supplementary Information:**

The online version contains supplementary material available at 10.1186/s12889-021-12003-0.

## Background

The ambitious goal to halve the number of global deaths and injuries from road traffic crashes by 2020 (United Nations Sustainable Development Goal 3.6) [1] has not been achieved, despite some improvement in higher income countries [2]. The most vulnerable road users, including motorcyclists and bicyclists, still account for over half of all road traffic deaths in recent global estimates [3, 4]. Road crash fatality rates in Australia have declined in vehicle occupants at around three times the rate than that for vulnerable road users in the past decade [5]. International studies demonstrate similar findings [4, 6]. Despite motorcycles comprising less than 5% of all registered motor vehicles in Australia [7], motorcycle riders represent more than one quarter of non-fatal hospital admissions [8, 9]. Similarly, motorcycles in the United States comprise around 3% of all registered vehicles, yet of motor vehicle traffic crashes in 2018, fatalities for motorcyclists were nearly 27 times more frequent than occupants in four-wheeled vehicle, and nearly four times more likely to be injured [10]. Motorcyclists are therefore greatly over-represented in road trauma [5]. While cyclists are also a high-risk group as vulnerable road users [11, 12], this paper will focus on motorcyclist injuries and outcomes, with reference to other road users including cyclists.

A leading cause of serious injury and death among motorcyclists is head injury which is addressed legislatively in many countries [13, 14]; best practice standards mandating the wearing of fastened helmets on all roads, for motorcycle riders and passengers. Additionally, protective clothing has resulted in lower injury severity and been associated with some mitigation of post-crash health and well-being consequences in the immediate post injury phase [15]. Improved pre-hospital and trauma care have also significantly improved survival after land transport crashes [16]. Therefore, prevention efforts have shifted to focus on reducing the burden of the resultant disability and improving quality of life [17].

There are limited studies investigating longer-term outcomes in the vulnerable road user group of motorcyclists. One prospective cohort study of injured motorcyclists in Australia found that over half were still reporting pain at 6 months post-crash [15]. Another cohort study evaluating the psychological and economic impacts of motorcycle crash across three European countries reported similar findings [18], including markedly lower quality of life compared with pre-injury status. The authors concluded a need for longitudinal studies to explore in more detail factors that may influence long term effects of a road crash on injured motorcyclists’ well-being [18].

Injury related pain after a land transport crash can become chronic, interfering with daily functioning, and at times leading to substance dependence [19]. Few studies have examined the impact of pain severity after land crash injury. Gopinath et al. [20] showed that close to 20% of people injured in motor vehicle crashes report ongoing significant pain even 12-months after non-catastrophic injury. Factors identified as predictive of ongoing pain in that study included age, gender, self-rated health status, tertiary qualification, making a compensation claim and reporting neck or back pain or injury. Being a bicyclist was a protective factor against ongoing and more severe pain [20]. Motorcyclists were included but not specifically studied in that research [20]. Greater understanding of the factors associated with the burden of chronic pain, reduced functional capacity and poor quality of life in injured motorcyclists is vital, to inform post-crash treatment and care.

The Global Burden of Disease 2019 report estimates negligible reduction in rates of years lived with disability following injury since 1990, for countries with high socio-economic index like Australia [17]. Notably, the disability related to mental and substance-use disorders following injury is making increasingly large contributions to the global injury burden and related health expenditure, while depressive disorders are now among the ten key drivers of this growing burden [17]. Post-crash psychological distress may range from symptoms of depressed mood or elevated anxiety and can lead to disorders such as depression and Post-Traumatic Stress Disorder (PTSD) [18, 21]. Resultant ongoing psychological disability following injury can hinder the individual’s return to work, reduce capacity to maintain productive employment and engagement compared to pre-injury levels of social participation. Particularly, participation in society is strongly linked with improved psychological outcome after injury [22].

A clearer understanding of the longer term risks of poorer physical and psychological health recovery specific to road user groups after land transport crash injury will contribute towards the international priority of improvement of post-crash care support [23]. The importance of early, comprehensive identification of psychological trauma on injured individuals, and risk targeted intervention to mitigate the long-term impacts of depression and anxiety has been quantified [24], yet remains inconsistently applied in current practice [25]. Given the evidence of significant differences in longer term outcomes between different road user groups [20], yet insufficient investigation into motorcyclist outcomes among these comparisons, it is important to substantiate the factors that may hinder successful recovery in this population of vulnerable road users. Therefore, the aim of this study was to clarify how motorcyclists recover after a road traffic related injury (6 and 12-month post-injury), compared with other road user groups, namely bicyclists and vehicle occupants, in longitudinal comparison over the period from baseline to 12 months. We examined the differences between road user types before and after injury, considering the impacts of sociodemographic and pre-injury characteristics, and identified predictors significant to longer-term physical and psychological health outcomes.

## Methods

### Study population

A prospective inception cohort study design was used, the inception period defined as ‘within 28 days of injury.’ This period provided study site staff adequate time to identify and contact individuals admitted to hospital following land transport crash, recruit and interview them for baseline data collection. Almost half the participants were recruited from two major trauma hospitals in metropolitan Sydney, and the remainder from other participating hospitals in regional New South Wales (NSW). A small proportion (5%) were recruited from non-hospital sources (Personal Injury Register database maintained by the State Insurance Regulatory Authority, the Claims Advisory Services, and a small number from physiotherapists and/or GPs). The study period was from July 2013 to November 2016. Research nurses at each hospital site screened Emergency Department databases to identify potential study participants.

Inclusion and exclusion criteria have been previously reported in detail [26]. Briefly, individuals were eligible for inclusion if they were a) aged ≥17 years b) had experienced a land transport crash in NSW between July 2013 and November 2016, and c) had been diagnosed with physical injury because of this crash by a registered health practitioner within 28 days of this crash. Consenting individuals meeting inclusion criteria were identified and invited to participate in the study. Injury severity for the purposes of this study, was defined using the injury severity scale scores (ISS) [27] and ISS scores were derived by a trained coder. The ISS scores were calculated using the Abbreviated Injury Scores (AIS) [28], specified for all injuries for 51.3% of records or relying on existing ISS scores for 12.1% of records. Text data were also searched and coded by the trained coder for 34.7% of records. Less than 1% of records showed discrepancies between the ISS and AIS scores; in these cases, standardised calculations were applied to obtain the lowest feasible ISS. Where there was no injury information available (0.4%), an ISS of 1 was assigned. The study protocol was approved by a Sydney Local Health District Human Research Ethics Committee (HREC/13/CRGH/67approval number).

### Baseline data

Data collected at baseline included participant socio-demographics, nature of the injury sustained (self-reported) and consequent hospitalisation (length of stay in hours dichotomised as spending < 12 h or ≥ 12 h in hospital), pre-injury comorbidity including 18 specific conditions (Appendix A), pre-injury physical comorbidities from a list of 16 (Appendix B), pre-injury history of depression or anxiety or panic disorder, pre-injury work status and income bracket, highest level of education completed, self-reported anthropometric data (height and weight) from which was derived body mass index (BMI). BMI was calculated by dividing participants’ weight (in kilograms) by their height (in metres squared) [29]. Substance use including cigarette smoking and alcohol consumption was also self-reported. The 3-item Audit-C screen [30] was used to determine the extent to which a participant’s alcohol use affected his/her safety; scores on the Audit-C range from 0 to 12, with higher scores indicating higher effects on safety [31]. The Index of Relative Socio-economic Advantage and Disadvantage (IRSAD) was used to measure socioeconomic status based on an individual’s residential postcode [32]. The IRSAD scores were analysed by in deciles; the lowest decile indicating the most disadvantaged neighbourhoods and the highest decile indicating the most advantaged neighbourhoods. Mean (SD) scores by road user group were reported.

### Assessment of psychological and health status

For baseline and follow-up assessment of psychological status and impact, a variety of outcome measures were used. Participants were asked to respond to the Depression, Anxiety and Stress Scales short-form (DASS-21). This is a validated self-report psychometric tool designed to measure the negative emotional states of depression, anxiety and stress. It is scored from 0 to 63 where higher scores indicate higher levels of symptoms [33]. The Impact of Event Scale – Revised (IES-R) was also used, being a validated measure of post-traumatic stress symptoms. It is scored 0–12 where higher scores indicate higher stress [34]. Participants also completed the EuroQol 5D-3L (EQ-5D) [35, 36], a validated tool used to measure health related quality of life comprising of five dimensions: mobility, self-care, usual activities, pain/discomfort and anxiety/depression, each of which has three levels of response (no problems, some problems, extreme problems/am unable to). Each dimension scores 1 (indicating no problems) to 3 (indicating extreme problems) and produces a numeric health state. We used the summary index derived from the time trade-off valuation technique [37]. Participants also reported scores on the Visual Analogue Scale (VAS) (scored with the EQ-5D), as a response from 0 to 100, where 0 means the worst health imaginable by the respondent, on the day of completion; 100 means the best health imaginable by the respondent, on the day of completion. The Short Form 12 (SF-12) is a brief, validated instrument for measuring health related quality of life. The SF-12 mental component scale (SF-12MCS) and physical component scale (SF12-PCS) were used to assess eight health domain outcomes. The MCS and PCS scores are within the range from 0 to 100, where higher scores are better [38]. Participants rate their pain at the time of survey completion on a scale from 0 to 10 where higher scores indicate higher levels of pain. No pain was scored as 0 out of 10. The pain catastrophizing scale was used to enable qualification of an individual’s perceived threat value of the pain stimulus and was scored from 0 to 52 where higher scores indicate higher levels of perceived threat [39].

### Definitions

For the purposes of this study, motorcyclists were defined as both riders and pillion passengers, and no restriction was placed on motorbike type or engine capacity for inclusion. Return to work or education status (scored as yes/no) was measured among those who were in work or studying prior to their injury. Work status was defined based on reported current paid work or self-employment across all participants. Compensation claims post-crash were identified within the government regulatory authority’s Personal Injury Registry database; defined by the presence or absence of a Compulsory Third Party (CTP) insurance claim.

### Statistical analysis

Statistical analyses were performed using SAS, version 9.4. Baseline characteristics of motorcyclists, bicyclists and vehicle occupants in the cohort were summarised using descriptive statistics. Differences in study characteristics were compared using the χ^2^-square test, general linear model F tests, or t-tests where appropriate.

Linear mixed regression models for repeated measures [40] were used to separately examine associations between road user type and each individual outcome measure (DASS-21, IES-R, EQ. 5D-3L, SF12-MCS, SF12-PCS; including their baseline, 6 month and 12 month values [40]. For the dichotomous paid work outcome, a generalised linear mixed model with the binomial distribution was used. After the mixed model was run, difference between road user types were examined at each individual time point. This can be referred to as an analysis of simple effects. Potential adjustment factors were considered under a causal inference framework using directed acyclic graphs. Models were first run with adjustments for a minimally sufficient set of common antecedent factors of both road user type and outcome identified using DAGGITY software (age group, gender, recruitment source, number of pre-injury comorbidities, pre-injury disability, pre-injury history of anxiety or depression). They were then run again after additional adjustments for CTP claimant status, baseline psychological status (IES-R, DASS21, pain catastrophizing), crash and injury factors (perceived danger in the accident, hospital length of stay, baseline pain) and two additional pre-injury factors (pre-injury paid work, pre-injury social satisfaction). The model interaction term for ‘time point by road user type’ was used to test whether effects of road user type on the relevant outcome measures differed significantly between the 12-month time point and the baseline post-injury time point.

Within the motorcyclist sample, linear mixed models with data from baseline through to 12-months were also used to examine a broader range of predictive factors for SF-12PCS and MCS outcomes at 12-months. Results are presented in terms of an analysis of simple effects for each predictive factor at 12 months post-injury. Each outcome was modelled separately. Effects of each predictive factor were evaluated after adjusting for factors which precede or arise in conjunction with the explanatory factor of interest. This approach to adjustment chooses adjustment factors theoretically, based on principles for causal inference. Thus, effects of pre-injury factors (recruitment source, age group, gender, pre-injury paid work or self-employment, pre-injury social satisfaction, pre-injury educational level, pre-injury physical comorbidities, pre-injury anxiety, or depression, pre-injury EQ-5D summary score) were evaluated after adjustment for all other pre-injury factors. Effects of crash and injury factors (perceived danger of death in the accident, initial hospital length of stay, baseline pain) were evaluated after adjustment for all other pre-injury and crash/injury factors. Effects of baseline psychological factors (IES-R, DASS21, pain catastrophising) and CTP claimant status were evaluated after adjusting for pre-injury factors, crash and injury factors, and other baseline psychological factors. Where effects were identified, the presence of associations at the initial baseline post-injury time point and the impact of additional adjustments are also noted.

## Results

Primary analysis involved 1854 participants who were recruited at baseline, in three road user groups. Motorcyclists comprised 33.9% of the overall study cohort of injured road crash participants (*n* = 628), with 299 (16.1%) bicyclists and 927 (50%) vehicle occupants recruited at baseline during the study period. Substantially more injured motorcyclists were male (88.1%) than injured bicyclists (75.9%; *p* < 0.001) and almost double that of vehicle occupants (46.7%), and motorcyclists were younger on average (38 years) than bicyclists (41.5 years) (Table 1). Motorcyclists were much less likely to have a tertiary education than both other road user groups (29% vs vehicle occupants 35.4% and bicyclists 66.6%. *p* < 0.001) and had lower incomes overall; with bicyclists predominantly reporting the highest socio-economic status (IRSAD mean (SD) for bicyclists 9.1 (1.7) vs motorcyclists 6.9 (3.0) and vehicle occupants 6.6 (3.2), *p* < 0.001). Motorcyclists were more likely to report being ‘never married’ (44.3%) than vehicle occupants (34.9%) or bicyclists (37.1%)(*p* < 0.001). Pre-injury substance use behaviour identified motorcyclists and vehicle occupant groups as significantly more likely to smoke than bicyclists (p < 0.001), and motorcyclists and bicyclists had higher average AUDIT-C scores (mean alcohol intake) than vehicle occupants (*p* < 0.0001). Pre-injury co-morbidity (Appendix A and B) was highest among vehicle occupants, who also had highest levels of pre-injury mental health concerns (27.9%, *p* < 0.001). Table 1 describes the baseline sociodemographic, health and crash related characteristics, comparing them between the three road user groups in this study. Comparisons are made between motorcyclists and vehicle occupants and then motorcyclists and bicyclists, with significant findings indicated as footnoted (* or **) in the comparator column.

**Table 1:** Baseline socio-demographic, health and crash-related characteristics for motorcyclists, vehicle occupants and bicyclists

	Motorcyclists (***n*** = 628)	Vehicle occupants (***n*** = 927)	Bicyclists (***n*** = 299)
	Mean (SD) or n (%)	Mean (SD) or n (%)	Mean (SD) or n (%)
**Age (mean, SD)**	38.0 (13.4)	43.2 (18.8)^**^	41.5 (12.7)^**^
**Male gender**	553 (88.1)	433 (46.7)^**^	227 (75.9)^**^
**Country of birth**		**	*
Australia	490 (78.0)	621 (67.0)	210 (70.2)
New Zealand	19 (3.0)	20 (2.2)	13 (4.4)
United Kingdom	32 (5.1)	57 (6.2)	28 (9.4)
Other	87 (13.9)	229 (24.7)	48 (16.1)
**English as primary language**	590 (94.0)	813 (87.7)^**^	289 (96.7)
**Marital status**		**	**
Divorced/widowed/separated	47 (7.5)	129 (13.9)	11 (3.7)
Married or de facto	303 (48.3)	474 (51.2)	177 (59.2)
Never married	278 (44.3)	323 (34.9)	111 (37.1)
**Educational level**		**	**
Primary or pre-primary	41 (6.5)	67 (7.2)	6 (2.0)
Secondary	205 (32.7)	311 (33.6)	51 (17.1)
Technical or other further education	199 (31.7)	220 (23.8)	43 (14.4)
Tertiary or university	182 (29.0)	328 (35.4)	199 (66.6)
**Pre-injury paid work or self-employment**	535 (85.2)	627 (67.6)^**^	258 (86.3)
**Pre-injury income**		**	**
$0–20,799	21 (4.1)	45 (7.6)	7 (2.8)
$20,800–41,599	75 (14.8)	112 (19.0)	23 (9.3)
$41,600–64,999	147 (29.0)	183 (31.0)	43 (17.3)
$65,000–103,999	144 (28.4)	166 (28.1)	72 (29.0)
$104,000+	120 (23.7)	84 (14.2)	103 (41.5)
**SEIFA**^a^ **index (mean (SD) of IRSAD**^**b**^ **decile)**	6.9 (3.0)	6.6 (3.2)	9.1 (1.7)^**^
**Any pre-injury comorbidity on list of 18 specific items**^**c**^	314 (50.0)	595 (64.3)^**^	131 (43.8)
**Pre-injury physical comorbidities (list of 16)**^**d**^	262 (41.7)	497 (53.7)^**^	101 (33.8)^*^
**History of depression /anxiety/panic disorder**	134 (21.3)	258 (27.9)^**^	55 (18.4)
**BMI (mean, SD)**	26.6 (4.8)	26.7 (6.2)	24.8 (3.9)^**^
**Current smoking**	123 (19.6)	168 (18.1)	25 (8.4)^**^
**Alcohol intake - Audit-C score (mean, SD)**	3.91 (2.62)	2.60 (2.42)^**^	3.85 (2.35)
**Perceived danger of death**		**	**
Overwhelming	42 (6.8)	144 (15.8)	9 (3.1)
Great	84 (13.6)	171 (18.8)	30 (10.3)
Moderate	142 (23.0)	174 (19.1)	46 (15.8)
Small	139 (22.5)	148 (16.3)	73 (25.1)
None	211 (34.1)	272 (29.9)	133 (45.7)

^*^p < 0.05, ^**^p < 0.01 for the specific comparison of motorcyclists versus vehicle occupants and motorcyclists versus bicyclists

^a^*SEIFA* Socio-Economic Indexes For Areas ^b^ *IRSAD* Index of Relative Socio-economic Advantage and Disadvantage. Mean IRSAD of cohort displayed (10 = greater advantage, 1 = greater disadvantage)

^c^ Appendix A. ^d^Appendix B

Table 2 shows motorcycle crash participants were more frequently recruited from regional hospitals (27.7% vs vehicle occupants 10.6% and bicyclists 5.7%, *p* < 0.001), and bicyclist crash participants from metropolitan hospitals (86.3%, *p* < 0.001). It also shows that motorcyclists had the highest prevalence of hospital stay> 12 h (60.5% vs vehicle occupants 46.8% and bicyclists 41.8%, *p* < 0.001), longest mean (SD) length of hospital stay (3.34 (4.98) hrs vs vehicle occupants 1.9 (4.20) hrs vs bicyclists 1.39 (2.33) hrs, p < 0.001), and greater injury severity as measured by mean (SD) ISS score compared with other road user groups (4.5 (4.3) vs vehicle occupants 3.0 (3.2) vs bicyclists 3.9 (3.2), *p* < 0.001). Motorcyclists were more likely to experience lower extremity injuries (35% vs vehicle occupants 9.5% and bicyclists 15.3%, *p* < 0.001), and conversely less likely head/face injuries than vehicle occupants and bicyclists (2.7% vs vehicle occupants 8.9% vs bicyclists 13.2%, *p* < 0.001). Twice the proportion of vehicle occupants reported psychological injury in the crash compared with both motorcyclists and cyclists (31.8% vs motorcyclists 13.9% vs cyclists 14.4%, *p* < 0.001).

**Table 2:** Injury presentation, epidemiology and hospitalisation characteristics for motorcyclists, vehicle occupants and bicyclists

	Motorcyclists (***n*** = 628)	Vehicle occupants (***n*** = 927)	Bicyclists (***n*** = 299)
	*Mean (SD) or n (%)*	*Mean (SD) or n (%)*	*Mean (SD) or n (%)*
**Recruitment hospital**		**	**
RNSH^**a**^ or RPAH^**b**^	285 (45.4)	399 (43.0)	258 (86.3)
Orange, Dubbo, or Bathurst	174 (27.7)	98 (10.6)	17 (5.7)
Other	159 (25.3)	350 (37.8)	14 (4.7)
Non-hospital	10 (1.6)	80 (8.6)	10 (3.3)
**Psychological injury in crash**^**e**^	87 (13.9)	295 (31.8)^**^	43 (14.4)
**All body regions injured**^**e**^			
Head or face	108 (17.2)	325 (35.1)^**^	113 (37.8)^**^
Neck	80 (12.7)	505 (54.5)^**^	50 (16.7)
Spine or back	171 (27.2)	477 (51.5)^**^	71 (23.8)
Torso	256 (40.8)	495 (53.4)^**^	116 (38.8)
Upper extremity	424 (67.5)	581 (62.7)	249 (83.3)^**^
Lower extremity	451 (71.8)	386 (41.6)^**^	191 (63.9)^*^
**Area of predominant injury**^**e**^		**	**
Multiple areas	43 (7.3)	126 (14.8)	28 (11.6)
Head/face	16 (2.7)	76 (8.9)	32 (13.2)
Neck	9 (1.5)	154 (18.1)	5 (2.1)
Spine/back	30 (5.1)	74 (8.7)	10 (4.1)
Torso	129 (22.0)	210 (24.7)	49 (20.2)
Lower extremity	205 (35.0)	81 (9.5)	37 (15.3)
Upper extremity	154 (26.3)	130 (15.3)	81 (33.5)
**Hospital stay > 12 h**	380 (60.5)	433 (46.8)^**^	125 (41.8)^**^
**Length of stay (mean (SD))**	3.34 (4.98)	1.9 (4.20)^**^	1.39 (2.33)^**^
**Injury Severity Score (mean (SD))**	4.5 (4.3)	3.0 (3.2)^**^	3.9 (3.2)^**^
**Any ANF**^**c**^ **or CTP**^**d**^ **compensation claim**	179 (28.5)	393 (42.4)^**^	66 (22.1)^*^
**Any CTP**^**d**^ **compensation claim**	132 (21.0)	264 (28.5)^**^	47 (15.7)

^a^*RNSH* Royal North Shore Hospital (Major Trauma Service, Metropolitan)

^b^*RPAH* Royal Prince Alfred Hospital (Major Trauma Service, Metropolitan)

^**c**^ *ANF* Accident Notification Form. ^d^ *CTP* Compulsory Third Party. ^**e**^ Self-reported

^*^p < 0.05, ^**^p < 0.01 for the specific comparison of motorcyclists vs vehicle occupants and motorcyclists vs bicyclists

Table 3 describes paid work (including self-employment), pain experience and the health-related self-reported quality of life measures, at baseline and at 6- and 12-month follow-ups. Around 85% of both motorcyclists and bicyclists were in paid work prior to their injuries, a higher proportion compared to vehicle occupants (67.6%). However, when followed up, at 6 and 12-months, slightly more bicyclists than motorcyclists had returned to paid work, with both more likely to be working than vehicle occupants. Notably, motorcyclists and bicyclists reported similar levels of any pain at their baseline interview, with both having higher levels than car occupants. By 12 months following injury, motorcyclists were significantly more likely to report ongoing pain than bicyclists (*p* < 0.001); but around the same levels as vehicle occupants (*p* = 0.12).

**Table 3:** Descriptive statistics for return to work, pain, quality of life, and psychological outcome measures over a 12-month period in motorcyclists versus vehicle occupants, then motorcyclists versus bicyclists

	Pre-Injury* N (%)	Baseline post-injury N (%)*	6 months N (%)**	12 months N (%)***
**Return to work (among paid workers pre-injury)**				
Motorcyclists:		–	368 (90.2) ^**£**^	302 (90.2) ^**£**^
vs Vehicle occupants		–	341 (84.4) ^**£**^	281 (86.7) ^**£**^
*p value*			*0.01*	*0.17*
vs Bicyclists		–	209 (94.6) ^**£**^	186 (94.9) ^**£**^
*p value*	*0.6*		*0.057*	*0.053*
**Any pain**				
Motorcyclists:	Not available	561 (89.3)	324 (66.8)	227 (58.1)
vs Vehicle occupants	Not available	773 (83.4)	408 (66.0)	309 (63.2)
*p value*		*0.001*	*0.9*	*0.12*
vs Bicyclists	Not available	270 (90.3)	152 (58.4)	89 (38.9)
*p value*		*0.6*	*0.08*	*< 0.0001*
	**Mean (SD)**	**Mean (SD)**	**Mean (SD)**	**Mean (SD)**
**EQ-5D-3L VAS**				
Motorcyclists:	86.3 (12.4)	Not available	79.6 (14.6)	80.8 (14.9)
vs Vehicle occupants	84.5 (13.9)	Not available	75.1 (18.8)	75.8 (19.0)
*p value*	*0.01*		*< 0.0001*	*< 0.0001*
vs Bicyclists	86.8 (10.4)	Not available	82.4 (13.1)	83.0 (13.9)
*p value*	*0.5*		*0.03*	*0.11*
**EQ-5D-3L TTO**^**€**^ **summary score**				
Motorcyclists:	0.94 (0.12)	0.36 (0.37)	0.80 (0.21)	0.83 (0.22)
vs Vehicle occupants	0.91 (0.15)	0.42 (0.38)	0.73 (0.30)	0.78 (0.28)
*p value*	*< 0.0001*	*0.004*	*< 0.0001*	*0.0009*
vs Bicyclists	0.95 (0.10)	0.47 (0.33)	0.86 (0.20)	0.89 (0.19)
*p value*	*0.4*	*< 0.0001*	*0.002*	*0.006*
**SF12-PCS**				
Motorcyclists:	Not available	31.9 (10.9)	47.0 (10.1)	48.9 (9.7)
vs Vehicle occupants		36.3 (11.9)	45.3 (11.2)	46.0 (11.2)
*p value*		*< 0.0001*	*0.008*	*< 0.0001*
vs Bicyclists	Not available	35.6 (11.0)	50.7 (8.1)	52.3 (7.0)
*p value*		*< 0.0001*	*< 0.0001*	*< 0.0001*
**SF12-MCS**				
Motorcyclists:	Not available	51.0 (11.8)	53.8 (9.5)	53.3 (9.8)
vs Vehicle occupants		46.8 (12.1)	49.8 (11.6)	51.0 (10.5)
*p value*		*< 0.001*	*< 0.0001*	*0.0006*
vs Bicyclists	Not available	52.3 (10.2)	53.2 (9.6)	54.8 (8.3)
*p value*		*0.12*	*0.5*	*0.07*
**IES-R total**				
Motorcyclists:	Not available	2.85 (2.74)	1.84 (2.50)	1.71 (2.58)
vs Vehicle occupants		4.5 (3.4)	3.1 (3.2)	2.5 (3.1)
*p value*		*< 0.0001*	*< 0.0001*	*< 0.0001*
vs Bicyclists	Not available	2.49 (2.24)	1.52 (2.13)	1.06 (1.78)
*p value*		*0.09*	*0.14*	*0.004*
**DASS21 total**				
Motorcyclists:	Not available	10.2 (12.6)	7.8 (12.2)	7.6 (12.6)
vs Vehicle occupants		15.5 (16.8)	13.1 (16.5)	10.3 (14.7)
*p value*		*< 0.0001*	*< 0.0001*	*0.002*
vs Bicyclists	Not available	8.0 (10.3)	6.7 (11.1)	4.8 (9.0)
*p value*		*0.03*	*0.3*	*0.01*
**Pain scale**^**&**^				
Motorcyclists:		4.3 (2.5)	2.1 (2.2)	1.8 (2.2)
vs Vehicle occupants		4.4 (2.9)	2.9 (2.9)	2.6 (2.7)
*p value*		*0.4*	*< 0.0001*	*< 0.0001*
vs Bicyclists		3.7 (2.4)	1.6 (2.0)	1.0 (1.7)
*p value*		*0.003*	*0.01*	*< 0.0001*
**Pain catastrophizing scale**^**&**^				
Motorcyclists:		12.0 (12.6)	7.3 (11.1)	6.6 (11.7)
vs Vehicle occupants		15.9 (15.2)	12.4 (15.3)	10.2 (13.9)
*p value*		*< 0.0001*	*< 0.0001*	*< 0.0001*
vs Bicyclists		9.7 (10.6)	5.7 (9.6)	3.4 (7.8)
*p value*		*0.02*	*0.13*	*0.002*

*Pre-injury N: Motorcyclists = 628, Vehicle occupants = 927, Bicyclists = 299

**6-month follow-up N (%): Motorcyclists = 485 (77.2), Vehicle occupants = 618 (66.7%), Bicyclists = 260 (87.0)

**12-month follow-up N (%): Motorcyclists = 391 (62.3), Vehicle occupants = 489 (52.8%), Bicyclists = 229 (76.6)

^**£**^for these specific cells only, the calculations are restricted to individuals who were in paid work at baseline

^**€**^TTO = Time Trade-Off valuation technique. ^#^VAS = Visual Analogue Scale. ^&^ (taken as 0 if no pain)

Motorcyclists reported the greatest immediate effects of the injury on physical and overall function on average, but by 12-month follow up they reported better physical and overall function than vehicle occupants, while worse than bicyclists (EQ. 5D, SF-12 PCS, pain). They displayed less evidence of psychological distress than vehicle occupants, but more than bicyclists (DASS21, IES-R, pain catastrophising).

Table 4 shows the findings from modelling the adjusted effects of crash role on various 12-month outcomes, using linear mixed models for repeated measures from baseline to 12-months. Our primary analyses adjust for a minimally sufficient set of antecedent factors (age group, gender, recruitment source, educational level, pre-injury health, pre-injury disability, pre-injury history of depression or anxiety). Sensitivity analyses for the inclusion of additional adjustment factors are shown in footnotes.

**Table 4:** Adjusted effects of being a motorcyclist versus vehicle occupant or a motorcyclist versus bicyclist on work, pain, quality of life and psychological outcomes

MOTORCYCLISTS VS VEHICLE OCCUPANTS (Reference category): Adjusted for minimally sufficient set of antecedent factors					
	Pre-injury		12 months		
	*Estimated odds ratio (95% CI)*	*P value*	*Estimated odds ratio (95% CI)*	*P value*	Interaction p value *
In paid work	1.5 (1.1, 2.1)	0.009^a^	1.4 (1.02, 2.1)	0.06	0.7
	**Baseline**		**12 months**		
*Outcome*	*Estimated mean difference (95% CI)*	*P value*	*Estimated mean difference (95% CI)*	*P value*	Interaction p value *
SF12 PCS	−5.21 (−6.46, −3.96)	< 0.0001^a^	2.07 (0.77, 3.36)	0.002^a^	< 0.0001^a^
SF12 MCS	2.49 (1.27, 3.71)	< 0.0001	1.01 (−0.29, 2.32)	0.12	0.03^a^
EQ 5D summary score	−0.09 (−0.13, −0.05)	< 0.0001^a^	0.03 (−0.01, 0.06)	0.09	< 0.0001^a^
Numeric pain scale	0.24 (−0.05, 0.54)	0.1	−0.51 (−0.83, − 0.2)	0.001^a^	< 0.0001^a^
DASS21 total score	−3.73 (−5.31, −2.15)	< 0.0001	−2.4 (−4.1, − 0.7)	0.005	0.07
IES-R total score	−1.31 (−1.65, − 0.98)	< 0.0001^a^	− 0.77 (−1.13, − 0.42)	< 0.0001	0.0002^a^
Pain catastrophising scale	−2.76 (−4.27, −1.25)	0.0003^a^	− 3.26 (− 4.89, −1.63)	< 0.001^a^	0.5
**MOTORCYCLISTS VS BICYCLISTS (Reference category):** Mean difference adjusted for minimally sufficient set of antecedent factors					
	**Pre-injury**		**12 months**		
*Outcome*	*Estimated odds ratio (95% CI)*	*P value*	*Estimated odds ratio (95% CI)*	*P value*	Interaction p value *
In paid work	1.4 (0.88, 2.2)	0.15	0.95 (0.55, 1.7)	0.8	0.14
	**Baseline**		**12 months**		
*Outcome*	*Estimated mean difference (95% CI)*	*P value*	*Estimated mean difference (95% CI)*	*P value*	Interaction p value *
SF12 PCS	−2.61 (−4.23, −0.99)	0.002^a^	−2.33 (−3.90, − 0.76)	0.004	0.7
SF12 MCS	−0.99 (−2.56, 0.58)	0.2	−0.97 (− 2.55, 0.61)	0.2	0.9
EQ 5D summary score	−0.08 (− 0.13, − 0.02)	0.002	−0.02 (− 0.06, 0.03)	0.4	0.014^a^
Numeric pain scale	0.27 (−0.11, 0.65)	0.16	0.47 (0.09, 0.84)	0.014	0.3
DASS21 total score	1.03 (−0.99, 3.06)	0.3	1.47 (0.6, 3.54)	0.16	0.6
IES-R total score	−0.11 (− 0.31, 0.54)	0.6	0.35 (− 0.08, 0.78)	0.1	0.2
Pain catastrophising scale	−0.92 (−1.03, 2.86)	0.3	1.72 (−0.25, 3.71)	0.08	0.4

*P value for interaction between road user group and time point

^a^ = Difference still found after additional adjustments for factors listed: pre-injury paid work, social satisfaction, perceived danger in accident, hospital length of stay, baseline pain and psychological status and CTP claimant status

Compared with vehicle occupants at the baseline post-injury assessment, motorcyclists had poorer physical health (SF-12 PCS) and overall health (EQ. 5D summary) scores, but better psychological health scores (IES-R and pain with catastrophising), after adjusting for pre-injury, crash/injury and baseline factors, and CTP claimant status. They also had lower baseline SF-12 MCS and DASS21 scores than vehicle occupants when other baseline and post-injury factors were omitted from the adjustment model. However, after 12-months, motorcyclists had recovered physical health better than vehicle occupants and reported less pain and less pain with catastrophising after adjusting for pre-injury, crash/injury and baseline factors and CTP claimant status. They continued to report less ongoing psychological distress (DASS21 and IES-R) on average after adjusting for pre-injury and crash/injury factors but not after further adjustment for all aspects of baseline post-injury status and CTP claimant status.

Compared with bicyclists at baseline post-injury assessment, motorcyclists had poorer physical health (SF-12 PCS) and overall health (EQ. 5D summary) scores but had similar mental and psychological health. However, after 12 months, motorcyclists still had slightly poorer physical health than bicyclists (SF-12 PCS). They also had higher pain scores when other baseline and post-injury factors were omitted from the adjustment model.

Tables 5 and 6 show modelling results for adjusted effects of a variety of pre-injury, crash or injury, and baseline predictive factors for long-term SF-12 PCS and MCS outcomes 12 months post-injury, within motorcyclists only. Results are derived from longitudinal linear mixed models. Our primary models included adjustment factors which either precede or arise in conjunction with each explanatory factor of interest. Response variables are not included in the models as explanatory factors. Sensitivity analyses on the effect of additional adjustments are noted via footnotes.

**Table 5:** Explanatory factors for SF12 PCS at 12 months post-injury within motorcyclists only

*Explanatory factor*	*Estimate (95% CI)*	*p value*	*Footnotes*
**CTP claimant status**	−6.3 (−8.4, −41)	*<.001*	*a*
**High baseline IES-R**	−6.3 (−8.5, −4.2)	*<.001*	*b*
**High baseline DASS**	−3.7 (− 5.6, − 1.7)	*0.003*	*b*
**High baseline PCS**	−10.4 (− 13.1, −7.6)	*<.001*	*b*
**Baseline history of pain after accident**	−5.3 (− 6.8, − 3.9)	*<.001*	*a,b*
**Hospital length of stay (severity)**			
Seven days or more	−9.5 (−12.1, −6.9)	*<.001*	*a,b*
Two-six days	−2 (−3.9, -0.1)	*0.04*	*a,c*
One day or less (reference)			
**Perceived danger in the accident**			*c*
Overwhelming	−4.5 (−8.2,-0.8)	*0.02*	
Great	−0.4 (− 3.3, 2.5)	*0.8*	
Moderate	−0.6 (−2.9, 1.7)	*0.6*	
Small	0.2 (−2.2, 2.5)	*0.8*	
None (reference)			
**Pre-injury anx/dep**	0.8 (−2, 3.5)	*0.6*	*a*
**Social satisfaction**			
Dissatisfied	−1.1 (−7.2, 5)	*0.7*	
Neither	− 1.2 (−4.9, 2.5)	*0.5*	
Satisfied (reference)			
**Recruitment source**			
Orange/Dubbo/Bathurst	1.3 (−0.9, 3.6)	*0.2*	*–*
Other hospitals	−0.6 (− 2.9, 1.8)	*0.6*	*–*
PIR/CAS/GP/Physio/Online	2.3 (−5.8, 10.5)	*0.6*	*–*
RNS/RPA (reference)			
**Paid work**	1.3 (−1.4, 4)	*0.3*	*–*
**Level of education**			*a*
Primary or less	−0.4(−5.2, 4.3)	*0.8*	
Secondary	−2.3 (−4.7, 0.1)	*0.05*	
Technical or other	−1.3 (−3.6, 1)	*0.3*	
Tertiary or university (reference)			
**Pre-injury disability on EQ. 5D summary score**			*a,c*
< =0.8	−4.2 (−7.2, −1.2)	*0.06*	
> 0.8–0.9	0.5 (− 2.4, 3.4)	*0.7*	
> 0.9- < 1	–		
1-full score representing no problems (reference)			
**Pre-injury health - number of comorbidities**			*a,b*
Four or more	−8.1(−12.8, −3.3)	*0.08*	
Two or three	−3.6(−6.4, −0.8)	*0.01*	
One	0.9 (− 1.4, 3.2)	*0.4*	
None (reference)			
**Male**	1.9 (−1, 4.7)	*0.19*	*a*
**Age group**			*a*
17–24 years	2.6 (−0.2, 5.4)	*0.07*	
25–44 years	0.9 (−1.3, 3.1)	*0.4*	
45–59 years (reference)			
60–69 years	−1.7 (−5.9, 2.5)	*0.4*	
70+ years	1.8 (− 5.9, 9.4)	*0.6*	

*Footnotes:*

-Explanatory factors were individually assessed after adjusting for covariates which precede or coincide but not those which are likely to follow afterwards, i.e.:-Pre-injury factors were adjusted for all other pre-injury factors.

-Crash/injury factors (perceived danger and hospital stay) and baseline pain were adjusted for pre-injury factors and other crash/injury factors

-Baseline psych factors and CTP claimant status were adjusted for pre-injury factors, crash and injury factors, baseline pain and other baseline psych factors

a = significant difference seen at baseline time point

b = difference still found after further adjustment for all of the factors listed

c = not significantly different after further adjustment for all of the factors listed

**Table 6:** Explanatory factors for SF12 MCS at 12 months post-injury within motorcyclists only

*Explanatory factor*	*Estimate (95% CI)*	*p value*	*Footnotes*
**CTP claimant status**	−0.6 (− 2.7, 1.6)	*0.5*	*a*
**High baseline IES-R**	−5.4 (− 7.8, − 3.1	*<.001*	*a, b2*
**High baseline DASS**	−10.4 (− 12.4, − 8.4)	*<.001*	*a, b2*
**High baseline PCS**	− 2.6 (− 5.7,0.4)	*0.08*	
**Perceived danger**			
Overwhelming	−6.6 (−10.3, − 3)	*0. 03*	*a, c*
Great	−3.4 (− 6.4, − 0.5)	*0.02*	*a, c*
Moderate	−1.8 (− 4.1,0.5)	*0.12*	*a*
Small	−0.6 (−3, 1.7)	*0.6*	
None (reference)			
**Baseline history of pain after accident**	−0.6 (− 2.2,1)	*0.5*	
**Hospital length of stay (severity)**			
Seven days or more	−4.7 (−7.4, −2.1)	*0. 03*	*a, b1*
Two-six days	−2.8 (−4.7, − 0.8)	*0.005*	*b1*
One day or less (reference)			
**Pre-injury anx/dep**	−3.5 (−6.2, − 0.7)	*0.01*	*a, b2*
**Social satisfaction**			
Dissatisfied	−1.4 (−7.3, 4.5)	*0.6*	*a*
Neither	−1.9 (−5.4, 1.7)	*0.3*	
Satisfied (reference)			
**Recruitment source**			
Orange/Dubbo/Bathurst	0.4 (−1.8, 2.6)	*0.7*	*a*
Other hospitals	0.7 (−1.5, 3)	*0.5*	
PIR/CAS/GP/Physio/Online	5.2 (− 3, 13.4)	*0.2*	
RNS/RPA (reference)			
**Paid work**	2.7 (0.1, 5.3)	*0.04*	*b1*
**Level of education**			
Primary or less	−1.7 (−6.4, 2.9)	*0.4*	
Secondary	−1.6 (−3.9, 0.8)	*0.18*	
Technical or other	−0.3 (−2.5, 2)	*0.8*	
Tertiary or university (reference)			
**Pre-injury disability on EQ. 5D summary score**			
< =0.8	−3.1 (−6, -0.1)	*0.04*	*a, b1*
> 0.8–0.9	−2.6 (−5.4, 0.2)	*0.07*	*a*
> 0.9- < 1	–		
1-full score representing no problems (reference)			
**Pre-injury health - number of comorbidities**			
Four or more	−0.5 (−5.1, 4.1)	*0.8*	
Two or three	−3.3 (−6.1, − 0.5)	*0.02*	*b1*
One	−0.2 (−2.4, 2.1)	*0.8*	
None (reference)			
**Male**	1 (−1.7, 3.7)	*0.5*	*a*
**Age group**			
17–24 years	0.5 (−2.3, 3.2)	*0.7*	
25–44 years	−0.5 (−2.7, 1.6)	*0.6*	
45–59 years (reference)			
60–69 years	−0.4 (−4.5, 3.7)	*0.8*	
70+ years	3.1 (−4.2, 10.5)	*0.4*	

***Footnotes:***

- Explanatory factors were individually assessed after adjusting for covariates which preceded or coincided with the injury only: i.e., pre-injury factors were adjusted for all other pre-injury factors

- Crash/injury factors (perceived danger and hospital stay) and baseline pain were adjusted for pre-injury factors as well as other crash/injury factors

- Baseline psychological factors and CTP claimant status were adjusted for pre-injury factors, crash and injury factors, baseline pain and other baseline psychological factors

a = significant difference seen at baseline time point

b1 = difference still found after further adjustment for all factors listed *except* other baseline psychological factors

b2 = difference still found after further adjustment for all factors listed *including* other baseline psychological factors

c = not significantly different after further adjustment for all factors listed

### Explanatory factors for long-term SF12 PCS outcomes in motorcyclists

With reference to Table 5, high baseline IES-R, DASS or pain with catastrophising scores shortly after the injury, a history of pain at baseline shortly after the accident, prolonged initial hospital stay (≥7 days) and having two or more pre-injury comorbidities were each associated with poorer average SF12 PCS scores at 12 months post-injury after adjusting for pre-injury, crash, injury and baseline post-injury factors and CTP claimant status. Participants who undertook CTP claims had poorer SF12 PCS on average at baseline and at 12 months post-injury.

### Explanatory factors for long-term SF12 MCS outcomes in motorcyclists

With reference to Table 6, high baseline IES-R and DASS scores and a pre-injury history of anxiety or depression were each associated with poorer average SF12 MCS at 12 months, after adjusting for pre-injury, crash, injury and baseline post-injury factors and CTP claimant status. Initial hospital stays of 2 days or more, greater disability on EQ. 5D summary score (≤0.80) and two or more pre-injury comorbidities were also associated with poorer SF12 MCS outcomes at 12 months. Comparatively, being in paid work pre-injury was associated with better SF12 MCS outcomes at 12-months, after adjusting for all other factors in the table except baseline psychological status, but not if baseline psychological status was included in the adjustment model.

## Discussion

In this cohort study we found that road user types significantly differ in important characteristics, including pre-injury health status and self-reported recovery after injury. Motorcyclists were found to have poorer pre-injury health status and worse physical health status and more severe injuries just after injury, compared with vehicle occupants. Analysis using mixed models for repeated measures enabled examination of the effects of explanatory factors on various outcomes (including at baseline), to evaluate whether and how that effected changed over time. This analysis showed that motorcyclists had significantly better 12-month outcomes (physical and psychological outcomes) compared with vehicle occupants, despite significant differences in key characteristics at baseline. Bicyclists demonstrated the highest self-rated pre-injury health status, and they reported good recovery up to 12-month follow up, despite their vulnerability in the event of a crash. Motorcyclists had more severe injuries (with longer length of hospital stay than vehicle occupants), yet they appeared to recover better over the ensuing 12-months. Motorcyclists demonstrated a stronger psychological profile at baseline, which may have been a contributing factor.

We evaluated psychological outcomes using the self-reported SF-12 MCS, DASS21, and IES-R. The extent of recovery among motorcyclists at 12-months post-injury, was significantly greater among those whose baseline mental health was better (lower baseline DASS and PCS), who reported less pain post-crash and who had shorter length of stay (implying lower injury severity). Visser et al. [41] recently reported similar findings in a land transport crash cohort, where higher baseline scores for anxiety and depressive symptoms were predictive of poorer outcomes at 12 months post-crash. While Visser’s cohort included all road user types, they evaluated them as one group only, their conclusions, supported by our study findings, suggests that early screening could identify those at risk of poorer psychological outcomes.

Significantly lower 12-month physical health as indicated by SF-12 PCS scores?, was observed among motorcyclists with two or three (β − 3.6, *p* = 0.01) or four or more (β −8.1, p < 0.001) pre-injury comorbidities. Another Australian traumatic injury cohort also found that individuals living with one or more comorbidities prior to their orthopaedic injury were more likely to report lower post-injury health status compared with those living with no comorbidities prior to injury [42]. Compared with car occupants at baseline (adjusting for pre-injury, injury and baseline factors and CTP claimant status), motorcyclists had poorer physical health on average (SF-12 PCS) and overall health (EQ. 5D summary score). After 12 months, however, their average physical health recovery was markedly better than car occupants, including reporting less pain (*p* < 0.001).

Comparisons with bicyclists at baseline post-injury, revealed that motorcyclists had poorer physical health (SF-12 PCS) and poorer overall health (EQ. 5D summary score) but had similar mental and psychological health scores (after adjustment). After 12-months, motorcyclists still had slightly poorer physical health than bicyclists (SF-12 PCS) and had higher pain scores when other baseline factors were omitted from the adjustment model (β 0.4, *p* = 0.02). Gelaw et al. [42] reported their cohort of individuals with orthopaedic injury had better pre-injury self-reported health than the general population; this is supported by other studies and suggestive that people who experience trauma may over-rate or reconceptualise their pre-injury health status. The initially higher levels of pain reported by motorcyclists in this study, was lower than car occupants at 12-months post injury, but they had somewhat higher pain scores than bicyclists when other baseline factors were omitted from the adjustment model (β 0.4, *p* = 0.02). They also had lower pain catastrophising scores than car occupants when other baseline psychological and functional factors were omitted from the adjustment model. (β − 2, *p* < 0.0001). While motorcyclists in this study and other similar studies are predominantly male and younger, they typically have more severe injuries due to higher velocity crashes and are as such, more likely to develop persistent pain [43].

Overall, in this study, motorcyclists and bicyclists reported better quality of life outcomes, particularly across psychological measures, compared with vehicle occupants at 12-months following injury. This apparent “resilience” to land transport crash related injury among these two more vulnerable road user groups has also been recently reported in a large population registry-based cohort in Victoria [44]. A study involving a longitudinal follow up of over 5000 individuals following major road related trauma, found motorcyclists to have a 36% lower risk of reporting persistent problems and bicyclists a 60% lower risk [44]. Persistent problems were defined as ongoing anxiety/depression and/or pain/discomfort.

The surprising paradox of higher longer term pain levels and yet better reported mental health outcomes among motorcyclists has also been seen among a much larger Australian cohort of over 74,000 transport injury claimants, within which motorcyclists were found to have higher rates of pain but lower rates of mental health conditions compared with other road user groups [43], supporting other findings of lower risk of PTSD among motorcyclists compared with car occupants after a traffic crash [45]. While we are unable to provide causal interpretations of these findings in our study, this “resilience” reported by motorcyclists, despite the severity of crash related injury sustained, seems a consistent finding [44, 46] and worthy of further consideration.

The psychosocial factors influencing motorcycle rider’s behaviour, including sensation seeking, risk-taking and intentional safe riding has been the topic of previous research [47–50]. Tunicliffe et al. [50] studied a large cohort of Australian motorcyclists to analyse factors influencing rider behaviour, finding attitude and sensation seeking to be consistent predictors of risky riding intentions. While we did not question injured road users in this cohort as to their crash circumstances, previous studies have applied the Theory of Planned Behaviour to understand predictability of driver intention. For example, Elliot [51] showed that affective attitude, self-identify and perceived group norms among motorcyclists were positively correlated with intentions to speed. Tunicliffe [50] et al. also showed that social and self-identity among motorcyclists predicted riders’ intentions to engage in risky or risk averse on road behaviour, particularly noting that motorcycling is often undertaking as a group or social activity. In this study we found bicyclists to be more likely to be in married or de facto relationships and less likely to be divorced or separated than motorcyclists. Social and relationship structures have established significance in reducing risks of depression post injury [18], however, we did not explore the coping mechanisms identified by injured participants as substantive during their post injury recovery phase. This finding would be of interest and should be further explored. Considering the findings of our study in the context of other research into this road user group, cautious interpretation proposes motorcyclists to esteem the benefits of social inclusion and sensation seeking aspects of this activity, to substantially outweigh their vulnerability while doing so.

Our study had several limitations. Participants were not questioned regarding protective clothing or helmet wearing which would have impacted the severity of their injuries and may also complement information as to their risk-taking behaviours. Helmet wearing is mandatory in Australia, and enforcement results in high wearing rates, however protective clothing is worn at the rider’s discretion. Participants were also not questioned as to their driving/riding history. Years of driving experience and previous infringements are both influential variables in crash outcome studies. Previous studies have found that where older drivers ‘drive less’; this reduced mileage heightens their crash risk relative to younger age groups, and aligns with the older persons self-perceived ‘driving fitness’ [52]. However, our study did include age in all models, and there was a trend for older road users to report lower pre-injury EQ. 5D Visual Analog Scale (VAS) health ratings than younger road users. This relationship held within vehicle occupants and within motorcyclists but was reversed within bicyclists, whereby older bicyclists tended to have higher mean EQ. 5D VAS health ratings than their younger counterparts. Finally, we did not ask about levels of care provided in the acute post-crash phase, and while we do not make assumptions that care was equal regardless of injury severity, all data were self-reported which would make care levels a highly subjective variable and as such best not included.

## Conclusions

In this study, pre-injury health status differed between road user types, thus, having influence on their longer-term outcomes. Notably, that despite indications of poorer pre-injury physical health status among motorcyclists, they reported higher average pre-injury mental health scores, compared with vehicle occupants. Despite having sustained more severe injuries, this group of vulnerable road users reported significantly better physical and psychological outcomes at 12-months post injury compared with vehicle occupants, including less reported pain. This perceived resilience supports findings of previous research. One of the main goals of post-injury care is to aid the patient to return to their pre-injury health status, where possible, through acute treatment and rehabilitation. Targeted early intervention such as inpatient referral to a clinical psychologist, particularly for individuals with evidence of poorer psychological well-being, could aid in longer term improved outcomes and should be prioritised in the acute setting. Understanding these differences provides important evidence to aid prevention efforts and post-crash care, and among motorcyclists, clarifying reasons for why this group has higher psychological resilience after traffic injuries, despite ongoing pain, is a matter for future research.

## Supplementary Information

**Additional file 1.**

## Data Availability

Study data is not available to external request due to participant consent not providing for this. The corresponding author can be contacted for any specific queries on the data that was analysed for this report.

## Abbreviations

AIS: Abbreviated Injury Scale
BMI: Body Mass Index
CTP: Compulsory Third Party Insurance
DASS21: Depression, Anxiety and Stress Scales short form
EQ-5D: EuroQol 5D-3L
IES-R: Impact of Event Scale-Revised
IRSAD: Index of Relative Socio-economic Advantage and Disadvantage
ISS: Injury Severity Scale
NSW: New South Wales
SEIFA: Socio-Economic Indexes For Areas
SF-12: Short Form 12
SF-12MCS: Mental Component Scale
SF-12PCS: Physical Component Scale
