# Supplementary material for: Comparison of physical and psychological health outcomes for motorcyclists and other road users after land transport crashes: an inception cohort study
Source: BMC Public Health. 2021 Nov 2;21:1983. doi: 10.1186/s12889-021-12003-0 (PMC8565041; doi:10.1186/s12889-021-12003-0)
Supplement: Supplementary file 1 — Additional file 1. [file 12889_2021_12003_MOESM1_ESM.pdf]

Appendix A – Pre-injury comorbidity

All participants were asked whether they had been told by a Doctor that they had any of the following conditions:

- 01 Arthritis (rheumatoid and osteoarthritis) ☐
- 02 Osteoporosis ☐
- 03 Asthma ☐
- 04 COPD (chronic pulmonary disease), ARDS (adult respiratory distress syndrome) or emphysema ☐
- 05 Angina ☐
- 06 Congestive heart failure or heart disease ☐
- 07 A heart attack (that is, myocardial infarction) ☐
- 08 Neurological disease (e.g multiple sclerosis or Parkinson's disease) ☐
- 09 Stroke or transient ischemic attack ☐
- 10 Diabetes type I or II ☐
- 11 Peripheral vascular disease ☐
- 12 Upper gastrointestinal disease (ulcer, hernia, reflux) ☐
- 13 Anxiety or panic disorders ☐
- 14 Depression ☐
- 15 Visual impairment (e.g. cataracts, glaucoma, macular degeneration) ☐
- 16 Hearing impairment (that is, very hard of hearing, even with hearing aids) ☐
- 17 Degenerative disk disease (that is, back disease, spinal stenosis, or severe chronic back pain) ☐
- 18 Obesity or a BMI of 30 kg/m2 and over ☐

Appendix B – Pre-injury subset of physical disorders

All participants had any pre-injury physical disorders counted from the following list:

- Arthritis (rheumatoid and osteoarthritis) ☐
- Osteoporosis ☐
- Asthma ☐
- COPD (chronic pulmonary disease), ARDS (adult respiratory distress syndrome) or emphysema ☐
- Angina ☐
- Congestive heart failure or heart disease ☐
- A heart attack (that is, myocardial infarction) ☐
- Neurological disease (e.g multiple sclerosis or Parkinson’s disease) ☐
- Stroke or transient ischemic attack ☐
- Diabetes type I or II ☐
- Peripheral vascular disease ☐
- Upper gastrointestinal disease (ulcer, hernia, reflux) ☐
- Visual impairment (e.g. cataracts, glaucoma, macular degeneration) ☐
- Hearing impairment (that is, very hard of hearing, even with hearing aids) ☐
- Degenerative disk disease (that is, back disease, spinal stenosis, or severe chronic back pain) ☐
- Obesity or a BMI of 30 kg/m2 and over ☐
